# Hyperthyroxinemia and Hypercortisolemia due to Familial Dysalbuminemia

**DOI:** 10.1089/thy.2020.0315

**Published:** 2020-11-05

**Authors:** Carla Moran, Christoph Seger, Kevin Taylor, Susan Oddy, Keith Burling, Odelia Rajanayagam, Louise Fairall, Anne McGowan, Greta Lyons, David Halsall, Mark Gurnell, John Schwabe, Krishna Chatterjee, Christopher Strey

**Affiliations:** ^1^ Wellcome Trust-MRC Institute of Metabolic Science, University of Cambridge, Cambridge, United Kingdom.; ^2^ Risch Laboratory Group, Lagerstrasse, Buchs, SG, Switzerland.; ^3^ Department of Clinical Biochemistry, Addenbrooke's Hospital, Cambridge, United Kingdom.; ^4^ Institute of Structural and Chemical Biology, University of Leicester, Leicester, United Kingdom.; ^5^ eSwiss Medical and Surgical Center, St. Gallen, Switzerland.

**Keywords:** familial dysalbuminemic hyperthyroxinemia, assay interference, discordant thyroid function tests, hypercortisolemia, albumin

## Abstract

A 23-year-old man and his grandmother with hyperthyroxinemia and hypercortisolemia were heterozygous for an *ALB* mutation (p. Arg218Pro), known to cause familial dysalbuminemic hyperthyroxinemia (FDH). However, serum-free cortisol levels in these individuals were normal and total cortisol concentrations fell markedly after depletion of albumin from their serum. We conclude that binding of steroid as well as iodothyronines to mutant albumin causes raised circulating cortisol as well as thyroid hormones in euthyroid euadrenal individuals with R218P FDH, with potential for misdiagnosis, unnecessary investigation, and inappropriate treatment.

## Introduction

Familial dysalbuminemic hyperthyroxinemia (FDH), a dominantly inherited condition due to circulating mutant albumin with altered binding affinity for thyroid hormones (TH), is a recognized cause of elevated serum thyroxine (T4) in euthyroid individuals. Heterozygous mutations in the gene (*ALB*) encoding the albumin molecule (R218H, R218P, R218S, R222I) change side chains of amino acids around a T4 binding pocket (site 1) in the protein, thereby reducing steric hindrance and increasing its affinity for iodothyronines (1–3). This T4 binding site in albumin is also known to interact with steroids *in vitro* (4). In this study, we report two individuals with FDH due to R218P mutant albumin who exhibited hypercortisolemia and hyperthyroxinemia, likely due to increased binding of steroid as well as iodothyronines to mutant albumin.

## Patient History

A 23-year-old male Swiss army recruit (P1), on no medication or supplements, was investigated after collapse during endurance training. He was tachycardic (pulse rate 100 beats/min), without other hyperthyroid features, and overweight (body mass index 31 kg/m^2^) without stigmata of Cushing's syndrome. His circulating total and free THs were elevated [total T4 > 300 nmol/L (normal range [NR] 65–181); free thyroxine (fT4) 77 pmol/L (NR 9–19.1); free triiodothyronine 6.1 pmol/L (NR 2.63–5.7)], but with normal thyrotropin (TSH) levels (TSH 1.4 mU/L). Measurements of serum total cortisol in P1, using either immunoassay (1610 nmol/L, NR 170–500) or by tandem mass spectrometry (MS) (1920 nmol/L, NR 280–650), indicated that he was also markedly hypercortisolemic (Table 1). His circulating 17-hydroxyprogesterone (17-OHP) [immunoassay 12.5 nmol/L (NR 1.9–6.5); MS 8.1 nmol/L (NR <5)], cortisone (175 nmol/L, NR 34–91), and 11-deoxycorticostrone (0.35 nmol/L, NR <0.25) levels were also raised.

**Table 1.** Biochemical Measurements in Patients

	ALB geno-type	Total T4 (nmol/L)	fT4 (pmol/L)	fT3 (pmol/L)	TSH (mU/L)	CBG (μg/mL)	Total cortisol (immunoassay) (nmol/L)	Total cortisol (LCMS) (nmol/L)	Urine-free cortisol (nmol/L)	Serum-free cortisol (nmol/L)
P1	R218P	>300 (69–141)	>80 (10–19.0)	10.9 (3.5–6.5)	3.9 (0.35–5.5)	14 (10–25)	1610 (145–619)	1920 (145–619)	342 (99–378)	16.7 (12.3–44.2)
P2	R218P	>300 (69–141)	>154 (10.5–21)	10.6 (3.5–6.5)	0.93 (0.35–5.5)	10 (10.5–16)	479 (95–462)	480 (95–462)	N/A	8.5 (11–38)

Reference ranges in brackets.

*ALB*, gene encoding the albumin molecule; CBG, corticosteroid binding globulin; fT3, free triiodothyronine; fT4, free thyroxine; LCMS, liquid chromatography-mass spectrometry; N/A, not available; T4, thyroxine; TSH, thyrotropin.

His grandmother (P2), who is otherwise well, was also hyperthyroxinemic with relatively high, late daytime, serum total cortisol levels (Table 1). His 61-year-old mother exhibited similarly abnormal thyroid biochemistry [TSH 1.00 mU/L (NR 0.3–4.3); fT4 50 pmol/L (NR 9.0–19.0)], but declined further investigation.

## Results

Both P1 and P2 were investigated further, either under clinical auspices or with informed consent as part of an ethically approved protocol (Cambridgeshire LREC 98/154). Elevated total and fT4 concentrations with normal T4 binding globulin levels (20.8 μg/L, NR 14–31) in P1 prompted an alternative TH binding protein abnormality to be considered. *ALB* sequencing showed that P1 and his hyperthyroxinemic maternal grandmother (P2) were heterozygous for a mutation (R218P) (Supplementary Fig. S1), known to cause FDH.

**FIG. 1.**
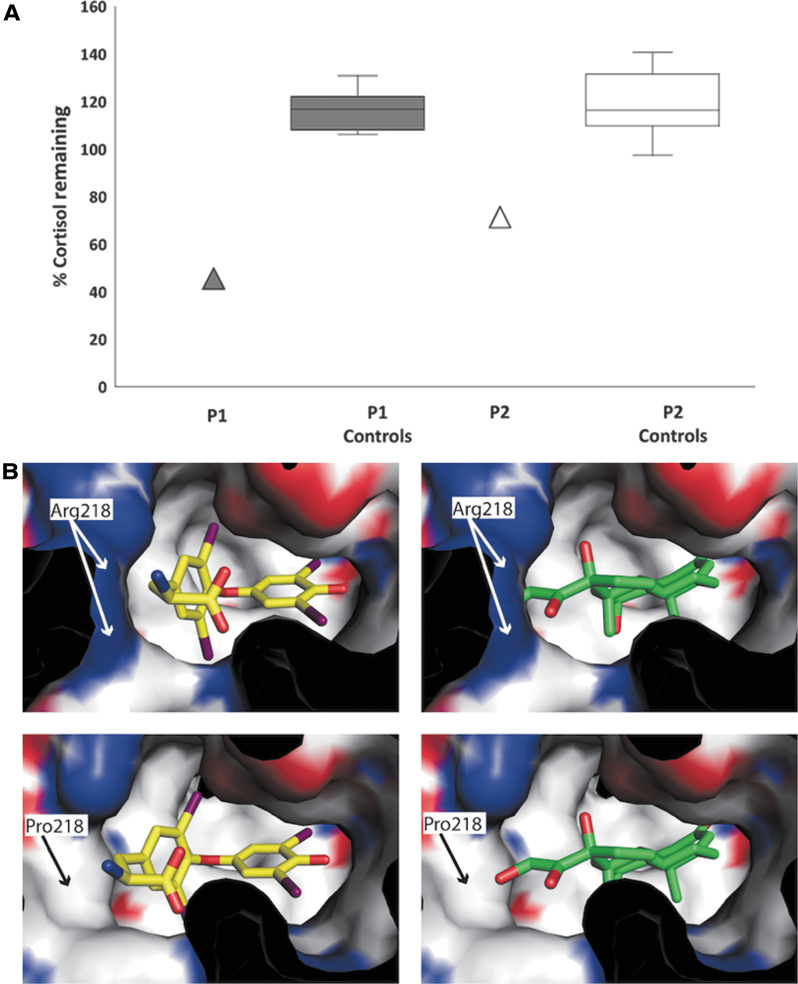
(**A**) Percentage of total cortisol remaining after immunodepletion of albumin from serum of individuals with R218P FDH (P1, P2) compared with that of age- and sex-matched healthy controls (*n* = 10). Box indicates interquartile range, horizontal line within box indicates median, and whiskers indicate range. (**B**) Top left: Structure of T4 bound to site 1 of wild-type albumin with the molecule positioned so that its polar ends are complementary with polar residues lining the binding pocket. Bottom left: Substitution of proline at position 218 reduces steric hindrance within the pocket, enabling T4 to be accommodated more easily while maintaining polar contacts. Top right: Cortisol, a longer molecule, is less easily accommodated within site 1 pocket of wild-type albumin. Bottom right: Substitution of proline at position 218 increases the width of site 1 pocket to accommodate cortisol easily. T4, thyroxine.

Given the discordance between hypercortisolemia and absence of features of Cushing's syndrome in P1, we measured free cortisol levels in his urine (342 nmol/L, NR 99–378) and serum (16.7 nmol/L, NR 12.3–44.2) and found them to be within the normal range (Table 1). We reasoned that his hypercortisolemia could be due to abnormal association of steroid with a circulating binding protein. Serum corticosteroid binding globulin (CBG) concentrations in P1 were normal (Table 1). However, after immunodepletion of circulating albumin from his serum, the total cortisol concentration fell markedly (to 46% of baseline) compared with minimal changes after albumin depletion of serum from age and sex-matched healthy control subjects (106–131%) (Fig. 1A).

His grandmother (P2) also showed discordance between relatively high, late daytime, serum total cortisol measurement (479 nmol/L) and low-normal serum-free cortisol (8.5 nmol/L, NR 11–38) concentrations (Table 1). Serum CBG concentration in P2 was normal (Table 1) but there was a greater fall in serum total cortisol (to 69% of baseline) after albumin immunodepletion than observed in healthy control subjects (97–141%) (Fig. 1A).

Modeling of cortisol binding to wild-type or mutant albumin indicates that the arginine to proline substitution at residue 218 reduces steric hindrance, enabling cortisol to be easily accommodated within site 1 pocket of the R218P mutant protein but not wild-type albumin (Fig. 1B). Serum cortisol levels in unrelated subjects with FDH due to other ALB mutations (R218H, R222I) were in the normal range (Supplementary Fig. S2). Methods are reported in Supplementary Data.

## Discussion

In two individuals with hyperthyroxinemia due to an *ALB* mutation (R218P) known to cause FDH, we have documented hypercortisolemia with euadrenal status and shown that this discordance is due to abnormal binding of steroid to circulating albumin.

A previous study supports this, showing that steroids (including corticosterone and 17-OHP) bind to site 1 T4 binding pocket of albumin (2). In this context, we note that, in addition to hypercortisolemia, P1 also exhibited elevated circulating 17-OHP, cortisone, and 11-deoxycorticosterone levels.

Modeling of R218P mutant albumin indicates that the reduction in steric hindrance from this amino acid substitution facilitates cortisol binding, providing a structural basis for our observations. Conversely, our finding of normal cortisol levels in individuals with R218H or R222I FDH (Supplementary Fig. S1) correlates with modeling showing that mutation of Arg218 to histidine or Arg222 to isoleucine would not increase the width of site 1 pocket to accommodate cortisol.

We have documented hypercortisolemia as well as hyperthyroxinemia in individuals with R218P dysalbuminemia, likely due to interaction of steroid as well as iodothyronines with mutant albumin, with spurious elevation of this combination of hormones, providing potential for misdiagnosis of an apparent new endocrine entity.

## Supplementary Material

Supplemental data
