# Supplementary material for: Hyperthyroxinemia and Hypercortisolemia due to Familial Dysalbuminemia
Source: Thyroid. 2020 Nov 5;30(11):1681–4. doi: 10.1089/thy.2020.0315 (PMC7692891; doi:10.1089/thy.2020.0315)
Supplement: Supplemental data [file Supp_Data.pdf]

Supplementary Data

Methods

Biochemical measurements

Hormone measurements were undertaken using automated immunoassay systems (free thyroxine, free triiodothyronine, thyrotropin, cortisol:Advia Centaur, TBG:Immulite; Siemens, Germany; Total thyroxine [T4]: AutoDelfia, Perkin Elmer, Belgium; 17-hydroxyprogesterone Roche) or by liquid chromatography mass spectrometry (cortisol, cortisone, 11-deoxycorticosterone). Serum corticosteroid binding globulin in patients (P1, P2) and controls (P1, 10 males age 18–23 years; P2, 10 females, age 57–65 years) was measured by ELISA (BioVendor).

Serum-free cortisol was assayed by overnight dialysis of serum at 37°C in buffer (S1), from patients (P1, P2) and controls (P1, 10 males age 18–23 years; P2, 10 females, age 57–65 years), with measurement of cortisol in dialysate by liquid chromatography-tandem mass-spectrometry as described previously (S2).

Cortisol was measured using liquid chromatography-tandem mass-spectrometry in serum samples from patients (P1, P2) and controls (P1, 10 males age 18–23 years; P2,

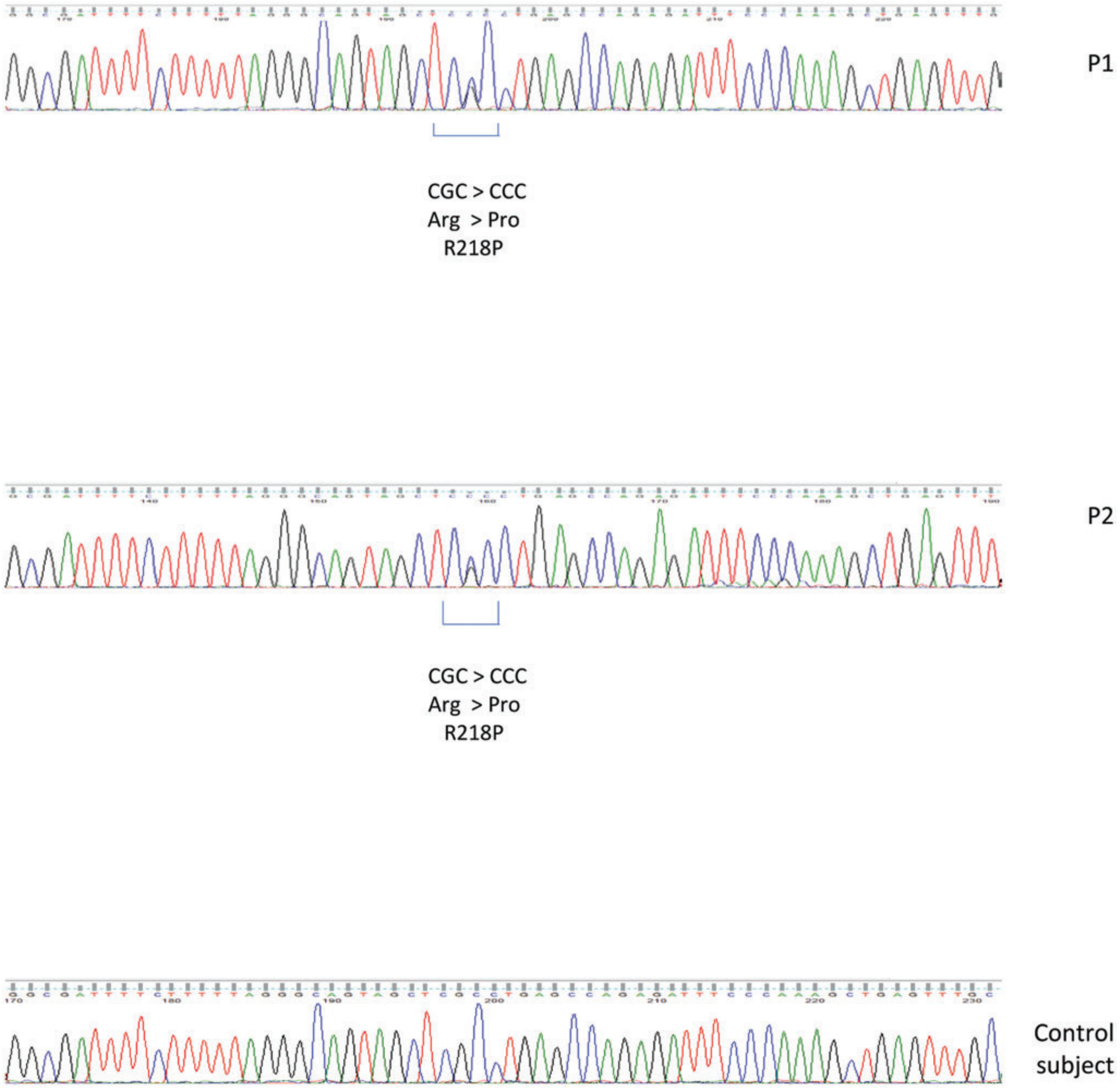

SUPPLEMENTARY FIG. S1. Electropherograms showing heterozygosity for a single nucleotide substitution (G/C) in the albumin gene (*ALB*) in P1 and P2, and wild type sequence from a healthy control subject.

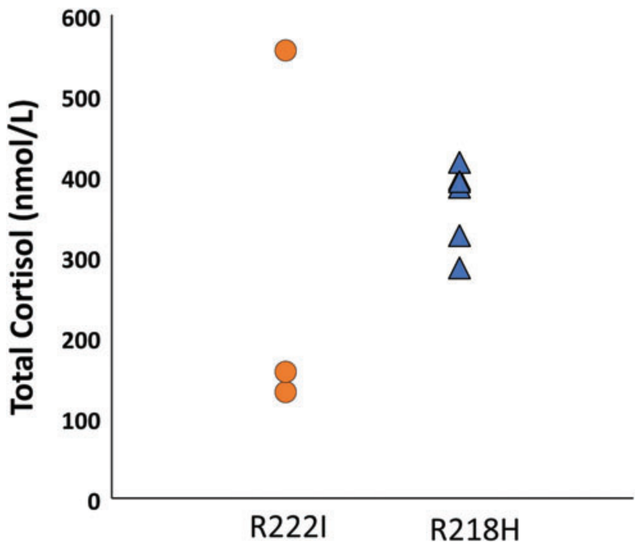

SUPPLEMENTARY FIG. S2. Total cortisol levels in serum from patients with R222I FDH and R218H FDH.

10 females, age 57–65 years) before and after depletion of albumin using antibody (PureProteome™ Albumin Magnetic Beads, Millipore, UK) as specified by the manufacturer. As the protocol involved significant dilution of samples, the results were adjusted for recovery of creatinine to correct for this.

Albumin gene sequencing

Exons of the human albumin gene were PCR amplified from genomic DNA using specific primers and analyzed by Sanger sequencing as described previously (S3).

Molecular modeling

Wild-type and mutant albumins were modeled (Pymol) using previously described wild-type albumin (1bm0) albumin-T4 (1hk1), R218H familial dysalbuminemic hyperthyroxinemia (FDH) mutant albumin-T4 (1hk2), and R218P FDH mutant albumin-T4 (1hk3) crystal structures (S4,S5), selecting the rotamer with the fewest clashes.

Supplementary References

S1. Nelson JC, Tomei RT 1988 Direct determination of free thyroxin in undiluted serum by equilibrium dialysis/radioimmunoassay. Clin Chem **34:**1737–1744.

S2. Kirchhoff F, Briegel J, Vogeser M 2011 Quantification of free serum cortisol based on equilibrium dialysis and isotope dilution-liquid chromatography-tandem mass spectrometry. Clin Biochem **44:**894–899.

S3. Schoenmakers N, Moran C, Campi I, Agostini M, Bacon O, Rajanayagam O, Schwabe J, Bradbury S, Barrett T, Geohegan F, Druce M, Beck-Peccoz P, O'Toole A, Clark P, Bignell M, Lyons G, Halsall D, Gurnell M, Chatterjee K 2014 A novel albumin gene mutation (R222I) in familial dysalbuminaemic hyperthyroxinaemia. J Clin Endocrinol Metab **99:**E1381–E1386.

S4. Petitpas I, Petersen CE, Ha C-E, Bhattacharya AA, Zunszain PA, Ghuman J, Bhagavan NV, Curry S 2003 Structural basis of albumin-thyroxine interactions and familial dysalbuminaemic hyperthyroxinemia. Proc Natl Acad Sci USA **100:**6440–6445.

S5. Sugio S, Kashima A, Mochizuki S, Noda M, Kobayashi K 1999 Crystal structure of human serum albumin at 2.5 A resolution. Protein Eng **12:**439–446.
